# The "Truth" Toy Fund

**Published:** 1886-12-04

**Authors:** 


### THE "TRUTH" TOY FUND.

Truth's Tender Side.

THAT man, it has been said, cannot be wholly bad who loves little children. The "Society Journal" which has distinguished itself by a ministry to sick and suffering little ones cannot be wholly given up to the delights of the tomahawk and the scalping-knife, however cynical and censorious it may commonly be considered to be. Such a ministry *Truth* has now been engaged in every Christmas since 1880, and, though this vivacious castigator of men and manners must since then have drawn down upon itself many a hearty malediction, it must also have evoked many a fervent blessing and many a kindly thought. I can answer for at least one particularly cordial appreciation of its Christmas bounty. I stood one Christmas morning by the beds of two pretty little cherubs, over whom relatives were dropping big tears, half in anguish half in delight. The day before they had rushed out from their house into one of the poorer streets of London, had been knocked down by a passing van, and both of them run over. Mangled and bleeding they were borne off to the hospital by the half-frantic parents, and the next morning I found them, badly hurt indeed, but not fatally or permanently as it had been feared, and both looking as though they considered themselves after all rather the favourites of fortune. Each had a rosy-cheeked doll and a bright new silver sixpence from the office of *Truth*, and very pretty it was to see the little sufferers' solicitude for the comfort of dolly, and their sparkling pleasure in the repeated unfolding of the shining coins, as they lay there in their pain and weakness. All through the women's wards of this general hospital I found little invalids thus gladdened, and if *Truth* had tomahawked my dearest enemy in its last issue, I think I could have forgiven it that Christmas morning.

The Start.

This genial enterprise began, as I have intimated, in 1880, when the Editor announced the offer of prizes to the value of four guineas, for the best home-made toys for distribution among the children in the various London hospitals. Many of the readers of the journal were, however, unable to make toys, and sent up subscriptions instead, and that first year about a thousand tiny patients had their Christmas morning brightened for them by the presentation of some sort of toy. It was a grand success. Next year the value of the prizes was increased to fifteen guineas, and on the memorable Christmas morning of 1881 every little inmate of a London hospital, altogether upwards of 1,500, had a toy presented, and in addition to this there were a few large toys—rocking-horses and so forth—sent to some of the hospitals for general use. Even after the distribution had taken place that year, subscriptions to the fund continued to be sent in, and with these the Editor purchased a dissolving view apparatus, with slides and all the requisite paraphernalia, to lend to hospitals and workhouses and any other institutions where there are children, whose lives need to be gladdened by a little bright and interesting entertainment. From that time to this that magic-lantern has gone on its beneficent way, and is now, I understand, about to have an addition to its attractiveness in the form of a lime-light apparatus.

Developments in 1882.

At the outset, the operations of the fund were confined to the wards of London hospitals, but in 1882 the means available appear to have out-run the requirements of the hospitals on Christmas morning, and it began to dawn upon the Editor that children in need of a little cheer and sunshine were not confined to hospitals; indeed, in many of these institutions there were, it was found, kindly souls who had made the supply of toys for the little sufferers their own peculiar care. Attention was turned to the workhouses, and workhouse schools and infirmaries—which of course are hospitals and in which a considerable number of children are always gathered. Their need was found to be quite as great, and the supply a good deal less, and on the Christmas day of 1882, 5,000 toys were dealt out to eager little hands in workhouses, and workhouse schools and infirmaries, as well as the public hospitals.

Model Management.

The fund was bound to be a success, especially when the public came clearly to understand that every penny of the money subscribed—without any deduction whatever—went in the purchase of the toys. The cost of actual distribution is indeed charged to the fund, but it is more than covered by *Truth's* own subscription to it; so that practically every sixpence sent is certain to buy a sixpenny toy for some little patient shut up in hospital, or immured within workhouse walls. The public took immensely to the idea, and in 1883 no less than 9,000 toys were exhibited in the large dining-room at Limmer's Hotel in Conduit Street, and afterwards distributed, and 5,000 sixpences were added to the gift by an anonymous donor; while among the old people of the workhouses, who had always shown a deal of enthusiastic enjoyment of the children's pleasure in the distribution, were dealt out little packages of tea for the old women, and snuff and tobacco for the old men. The next year there were 11,175 recipients of this pleasant Christmas bounty, which included 8,000 sixpences, and this Christmas, *Truth* tells us, the fund, which on the first occasion provided a gift for 1,000 children, has undertaken to scatter among the hospitals and charities, the workhouses, workhouse schools, infirmaries, and orphanages, upwards of 14,000 gifts, while all over London there will be screams of delight from throngs of little folks, this Christmas time, at the wonders and drolleries of that big magic lantern, and the music of fifteen large musical-boxes sent by a lady residing abroad. Experience, too, has shown how to make the best of the means at disposal, and next year the readers of this Journal are to have an opportunity of doll-dressing on a large scale. *Truth* will buy the dolls, of course, wholesale, and will deal them out in their unadorned simplicity to ladies who will undertake to return them in festive attire. Some arrangement of the kind has been adopted this year, but the dressing is being paid for. Next year the dressing is to be regarded as a contribution to the fund, and prizes are to be offered for the most successful toilets. No doubt many who could not make a toy, and perhaps some who are not in a position to contribute money to the fund, will gladly give their services in this way.

**Who'll Lend a Hand?**

It is a pleasant work altogether, and it is a pity every successful journal has not some philanthropic hobby of the kind. There are few indeed that would be likely to evoke so general an enthusiasm as this toy fund. A queer, cranky, embittered nature it must be, to which the sadness and suffering of childhood have no power of appeal, and which cannot respond with some little flutter of sympathy to a plea for a little brightness and pleasure at Christmas time for children on beds of languishing and pain, or doomed to the dull routine of workhouse life. It is not at all surprising that every year, as the operation of this fund has become more widely known, its supporters have become more numerous; but there is plenty of room for development yet. Hitherto, the Toy Fund has been confined to London as its sphere, and as we have seen, it has already pushed out far beyond the limits of the hospital ward. So far as all the greater hospitals are concerned, perhaps it may be said that enough is being done. In some of the more prominent and noted institutions for children, the tiny patients undoubtedly get a great deal of petting, and, if they happen to be pretty and interesting in any way, they are often in no little danger of being somewhat spoiled. But in the less prominent infirmaries and hospitals outside London, where they get comparatively few visitors, no press notices, no meetings and speeches to enlist the interest and sympathy of the kind-hearted public, there is still much to be done to lighten the load of sorrow and suffering, and dreary monotony and gloom that presses on many a sensitive little heart. Into many a minor country hospital *Truth's* Toy Fund might yet push its beneficent enterprise with a very cheery and joyful effect. Of course there are many country hospitals and infirmaries where much is done for the pleasure of children. Flowers and fruit in summer, and Christmas trees and toys and picture books in winter are by no means unknown. But there are without a doubt many institutions where there is a good deal less of this sort of thing than there ought to be, and the sympathetic public would everywhere do well to enquire a little into matters this Christmas, and if they see anywhere, in hospital wards or workhouse infirmaries, children whose lives are sad and dreary and wearisome, they should communicate with the Editor of *Truth*, who is quite ready to co-operate with his country readers in any effort they may think desirable for extending the operation of the Fund. If the public will find the money, *Truth* will gladly undertake to any extent to convert the money into toys, and to distribute them wherever there are eager little recipients to be found.

---

A Doctor's Bill.—A young M.D., having asked a pretty girl for a kiss, she caustically replied, "No, I thank you, I never like a doctor's bill stuck in my face."

